# A Novel Cryptococcal Meningitis Therapy: The Combination of Amphotericin B and Posaconazole Promotes the Distribution of Amphotericin B in the Brain Tissue

**DOI:** 10.1155/2020/8878158

**Published:** 2020-11-29

**Authors:** Ming Yang, Lin Cheng, Qing Dai, Bo Yang, Qian Yuan, MingJie Yu, Wei Feng, Fengjun Sun, Peiyuan Xia

**Affiliations:** ^1^Department of Pharmacy, The First Affiliated Hospital of Third Military Medical University (Army Medical University), Chongqing 400038, China; ^2^Department of Pharmacy, The Affiliated Hospital of North Sichuan Medical College, Nanchong 637000, China

## Abstract

The deficient brain tissue distribution of amphotericin B (AMPB) seriously restricts its treatment for the clinical efficacy of *cryptococcus neoformans* meningitis (CNM). We strive to develop a tactic to increase its concentration in brain tissue. We aimed to investigate whether the combination of AMPB and posaconazole (POS) could be more effective in the treatment of CNM and to elucidate its potential mechanisms. HPLC analysis was used to analyze the concentration of AMPB in mouse serum, brain tissue, and BCECs cells. Schrodinger molecular docking, in vitro plasma balance dialysis, and ultrafiltration analysis were performed to evaluate the combinative effect of AMPB and POS with serum albumin and POS on AMPB plasma protein binding. H&E staining and colonization culture experiment of CN were employed to assess the effect of POS on the efficacy of AMPB. POS + AMPB significantly reduced the concentration of plasma total AMPB and increased its concentration in the brain tissue. However, the P-gp inhibitor zosuquidar, BCRP inhibitor Ko143, and a common inhibitor of both, elacridar, had no significant effect on its concentration. Molecular docking, balance dialysis, and ultrafiltration analysis showed that AMPB and POS had potential binding properties to serum albumin. Meanwhile, 4 and 8 *μ*g/mL POS could significantly increase the concentration of free AMPB in plasma. POS and three inhibitors all had no significant effect on the uptake of AMPB by BCECs, but serum albumin had. The therapeutic effect of CNM in mice was confirmed that AMPB and AMPB+POS could restrain the infiltration of neutrophils and lymphocytes in cortical neurons and improve the bleeding and markedly inhibit the proliferation of CN. Collectively, we propose that POS competitively binds to the plasma protein sites of AMPB, thereby increasing its level in the brain tissue. Meanwhile, POS could enhance the efficacy of AMPB in the treatment of CNM, which may be independent of P-gp and BCRP proteins.

## 1. Introduction

As a kind of saprophyte bacteria, *cryptococcus neoformans*, also known as torula histolytica, is widely existing in natural soil, pigeon, milk, and fruit. For humans, it is an exogenous infection commonly by opportunistic pathogen [1]. Most of these fungal infections invadethe human lung tissue through the respiratory tract such as mouth, nasal cavity, and trachea causing mild pneumonia. On the other hand, it can enter the body from the damaged skin or intestines, causing damages to mucous membranes, lymph nodes, bones, and muscles and triggering chronic local inflammation and abscesses [2]. Critically, when the body's immune function declines, such as infection with human immunodeficiency virus or lymphoma patients, *cryptococcus neoformans* can invade the central nervous system (CNS), leading to a fungal infection in CNS, manifested as fungal meningitis, encephalitis, and granuloma of the brain, namely, cryptococcus neogenes meningitis (CNM) [3]. Its earlier clinical manifestations are irregular fever and intermittent headache with the characteristics of relatively insidious onset and long course. A small number of patients experience memory loss, personality changes, and restlessness. For patients with lower immunity, the symptoms are acute, headache, nausea, and vomiting [4].

Amphotericin B (AMPB) is a polyene antibiotic used in the treatment of invasive fungal infections and protozoosis. As two multidrug exlux transporters, P-glycoprotein (P-gp) belongs to the ATP binding cassette subfamily B member 1 (ABCB1), which is similar to the breast cancer resistance protein (BCRP) [5]. These two effervescent proteins are mainly expressed or distributed on the apical membrane of vascular endothelial cells, and their function is to prevent drugs or small molecular compounds in the blood vessels from entering tissues, such as the brain tissue and spinal cord. Therefore, both of them play a key role in regulating the functions of blood-brain barrier (BBB) and blood-spinal cord barrier (BSCB) and also affect the efficacy of tissue targeted drugs. Astrocytes have been shown to promote nuclear metastasis of NF*κ*B in vascular endothelial cells, and the resulting increased P-gp expression inhibits the distribution of drugs for treatment of amyotrophic lateral sclerosis (ALS) [6]. With the increase of age or the decrease of the P-gp protein expression in BBB vascular endothelial cells of patients with Alzheimer's disease (AD), the accumulation of neurotoxic amyloid-*β* (A*β*) may aggravate the pathological process of AD [7]. However, rivastigmine stimulated P-gp protein expression can enhance the extirpation of A*β* from BBB, finally showing excellent anti-AD efficacy [8]. For patients with schizophrenia, study has shown that long-term use of cannabinoid drugs, such as *Δ*^9^-tetrahydrocannabinol, can increase the P-gp protein expression in BBB. In response, the increased expression of P-gp would strengthen the brain efflux of the antipsychotic risperidone and its active metabolite, 9-hydroxy risperidone, while the efficacy of antipsychotics, such as clozapine, with non-P-gp protein substrates was not affected [9]. As a powerful means to probe P-gp, ^11^C-metoclopramide positron emission tomography (PET) imaging has confirmed the efflux effect of P-gp on the treatment of brain tumor drug metoclopramide [10]. Similarly, as an inhibitor of P-gp and BCRP, elacridar could inhibit the efflux of tyrosine kinase inhibitor erlotinib, resulting in improved efficacy against non-small-cell lung cancer (NSCLC) brain metastasis in nonhuman primate male Papio anubis baboons [11]. Further, evidence also suggested that P-gp and BCRP may reduce the brain distribution of tivozanib, a drug used to treat advanced kidney cancer [5]. Interestingly, studies have reported that AMPB may be the substrate of P-gp, evidenced by the increased AMPB distribution in the brain tissue by P-gp inhibitors verapamil and itraconazole [12]. On the contrary, uptake of AMPB by Caco-2 cells was independent of the P-gp expression; that is, AMPB is not the substrate of P-gp [13].

In view of the critical role of P-gp and BCRP in drug entry into the brain tissue and the current controversy, the purpose of this study was to investigate whether POS could promote the distribution of AMPB in the brain tissue to achieve better efficacy in the treatment of CNS and to elucidate its potential mechanisms.

## 2. Materials and Methods

### 2.1. Drugs and Reagents

AMPB (cat. no. MB1013-S) and POS (cat. no. MB1491) standards were purchased from Dalian Meilun Biotech Co., Ltd. (Dalian, China). AMPB liposome injection (lot. no. 181201) was obtained from Shanghai Shangyao New Asia Pharmaceutical Co., Ltd. (Shanghai, China). POS oral suspension (lot. no. M00801) was procured from Merck Sharp & Dohme (USA). Human albumin (lot. no. P100020500) was provided by CSL Behring (Switzerland). zosuquidar (cat. no. HY-15255), Ko143 (cat. no. HY-10010), and elacridar (cat. no. HY-50879) were purchased from MedChem Express (USA). Pancreatic enzyme (cat. no. SH30256.01), fetal bovine serum (FBS, cat. no. SV30087.03), 1640 medium (cat. no. SH30809.01), and mice plasma (cat. no. SH30042.01) were purchased from HyClone (USA).

### 2.2. Ethics

The production license number of experimental animals is SCXK (Yu) 2017-0002, and the use license number is SYXK (Yu) 2017-0002. The animals were kept in the laboratory animal observation room of the Third Military Medical University. All animal protocols involved in this experiment were approved by the Animal Research Ethics Committee of the Third Military Medical University.

### 2.3. Experimental Animal

A total of 60 male specific pathogen-free Kunming (KM) mice (weighing 18-22 g, aged 4 weeks old) were purchased from the Laboratory Animal Center of the Third Military Medical University. Mice were maintained in a 12 h light/dark cycle at room temperature (23 ± 2°C) in 50%-60% relative humidity during the whole experiment. The animals had ad libitum access to food and water, and the experiment was conducted after 7 days of adaptive feeding.

### 2.4. Grouping and Administration

30 mice were randomly divided into 5 groups (each, *n* = 6) as follows: AMPB group (3 mg/kg), AMPB (3 mg/kg)+POS (90 mg/kg) group, AMPB (3 mg/kg)+zosuquidar (20 mg/kg) group, AMPB (3 mg/kg)+Ko143 (10 mg/kg) group, and AMPB (3 mg/kg)+elacridar (2.5 mg/kg) group. The routes of administration of AMPB (q.d., 9 a.m), zosuquidar, Ko143, and elacridar were intravenous injection of the tail, and POS was administered by gavage. POS, zosuquidar, Ko143, and elacridar were administered for 4 consecutive days (b.i.d., 9 a.m. and 9 p.m).

### 2.5. HPLC Analysis of the Content of AMPB in Mice Plasma and Brain Tissue

4 h after the last administration, the mouse eyeball was taken for blood sample acquisition, in a commercial 1.5 mL centrifuge tube containing the anticoagulant EDTA, with sterilized ophthalmic bending forceps. Samples were centrifuged at 3000 rpm for 10 min. And the bottom plasma was gently extracted and separated with micropipeter. Then, plasma proteins were precipitated by adding 300 *μ*L methanol to 100 *μ*L plasma, followed by vortex for 10 s and let it stand for 5 min. After that, the samples were centrifuged at 4°C, 13000 rpm, for 10 min. Finally, the plasma test solution was fulfilled by filtering it through the 0.22 *μ*m microporous membrane.

After the completion of blood, the mice brain was quickly removed. Moderate brain tissue samples were mixed with physiological saline for homogenate preparation [14]. And proteins were precipitated by adding 300 *μ*L methanol to 100 *μ*L homogenate, followed by vortex for 10 s, and let it stand for 5 min, and then centrifuged at 4°C, 13000 rpm, for 10 min. Finally, the brain test solution was fulfilled by filtering it through the 0.22 *μ*m microporous membrane. The content of AMPB was determined by high-performance liquid chromatography (HPLC) using a Waters 2690 system (Waters, USA) at 405 nm [15]. The analytical column was a Diamonsil (2) C_18_ column (250 mm ×4.6 mm, 5 *μ*m) maintained at 30°C. The mobile phase consisted of 10 mM ammonium acetate aqueous solution (PH4) and acetonitrile, with the flow rate of 1.0 mL/min and the injection volume of 50 *μ*L.

### 2.6. Schrodinger's Molecular Docking Process

Referring to previous literature [16], serum albumin in the PDB format was downloaded from the Uniprot database (https://www.uniprot.org/). And for small molecule compounds, the 2D structures of AMPB and POS in the SDF format were download from the Pubchem database (https://pubchem.ncbi.nlm.nih.gov/). The receptor sites of serum albumin, the ligand sites of AMPB and POS, and all receptor-ligand docking procedures were determined and performed by Maestro 11.5 software. All the parameters involved are selected as software default settings.

### 2.7. Determination of the Concentration of AMPB in In Vitro Plasma by Balanced Dialysis and Ultrafiltration Analysis

The equilibrium dialysis experiments were performed by the Thermo Fisher Scientific rapid equilibrium dialysis (RED) device [17]. Appropriate sample solutions were prepared with mouse plasma at concentrations of AMPB 0.5 *μ*g/mL, AMPB 0.5 *μ*g/mL + POS 2.0 *μ*g/mL, AMPB 0.5 *μ*g/mL + POS 4.0 *μ*g/mL, and AMPB 0.5 *μ*g/mL + POS 8.0 *μ*g/mL. The prepared sample solutions were added into the holes of RED plate, respectively, with a 300 *μ*L sample loading. In the corresponding buffer holes, 550 *μ*L, 1% Triton X-100 solution was added. After the sample loading was completed, the sample was put into a 37°C constant temperature oscillator, and the dialysis was balanced at 100 rpm for 6 h. For ultrafiltration analysis, 1 mL of in vitro plasma was added into the centrifuge tube, and AMPB and POS of different concentrations were added according to the grouping details in Table 1, followed by mixing at 37°C for 2 h. And then, the protein or drug-protein complexes with a molecular weight ≥ 3000 kDa were retained by adding 1 mL of drug-containing plasma mixture into the Centrifree® overfiltration unit and centrifuging it at 13000 rpm and 4°C for 20 min. After the experiment, the concentrations of AMPB in dialysate and ultrafiltrate were determined by HPLC as described above.

### 2.8. HPLC Analysis of the Effect of POS on AMPB Uptake by Mice BCECs

The mice brain capillary endothelial cells (BCECs, cat. no. BNCC35187) were purchased from Beijing Beina Chuanglian Biotechnology Research Institute (Beijing, China). BCECs were resuscitated in a constant temperature water bath at 37°C and then inoculated in a petri dish [18]. The cells were cultured with the 1640 medium containing 10% FBS in a 5% CO_2_ cell incubator at 37°C. The culture medium was changed every other day, and the cells were digested with 0.25% trypsin when they converged and proliferated to form a full layer at the bottom of the dish. 1 mL cell suspensions were seeded in 12-well plates at a density of 1 × 10^5^ cells/mL per well. The drug was administered after the cells being completely adherent to the wall. 2.16 *μ*M AMBP solution was prepared with the 1640 medium and then was used to prepare POS solution at concentrations of 2.85 *μ*M, 5.71 *μ*M, and 11.42 *μ*M, 5 *μ*M zosuquidar, 5 *μ*M Ko143, 1 mM elacridar, and 40 g/L albumin solution, respectively.

The cells were randomly divided into the following 8 groups: 2.16 *μ*M AMBP, 2.16 *μ*M AMBP+POS (2.85 *μ*M, 5.71 *μ*M, and 11.42 *μ*M), 2.16 *μ*M AMBP+5 *μ*M zosuquidar, 2.16 *μ*M AMBP+5 *μ*M Ko143, 2.16 *μ*M AMBP+1 mM elacridar, and 2.16 *μ*M AMBP+40 g/L albumin. Three wells were set at each time point in each group and cultured at 37°C. Cell samples were collected at 30 min, 60 min, 120 min, and 180 min after drug administration, respectively. BCECs were eluted with Hanks' balanced salt solution for 3 times and then incubated with 1% Triton X-100 (200 *μ*L/per well) at 4°C for 12 h to lyse the cells. The cytolysis products were centrifuged at 4°C for 12,000 rpm for 2 min, and the content of AMPB was measured in the supernatant by HPLC as described above.

### 2.9. Efficacy of AMPB Combined with POS in Mice with Cryptococcal Meningitis

30 mice were randomly divided into 5 groups: control group, model group, AMPB (3 mg/kg) group, AMPB (3 mg/kg) + POS (180 mg/kg) group, and POS (180 mg/kg) group. Except the control group, the mice in the other four groups were all injected with the lateral ventricular cryptococcus suspension at the dose of 1 × 10^8^ CFU/mL, 5 *μ*L for each mouse [19]. And the control group was injected with the same volume of normal saline. A clinical strain of *Cryptococcus neoformans* was previously isolated from a patient with CNM and stored at the Chongqing Public Health Medical Center.

After 5 days, mice in each group were administered with the corresponding drugs mentioned above, continuously for 14 days. And the time and frequency of administration are described above. The mice were then anesthetized with ether for 24 h after the last administration. Next, the brain tissue was promptly removed for H&E staining and colonization detection of *Cryptococcus neoformans*.

### 2.10. Evaluation of Brain Pathology by H&E Staining

The mouse brain tissue was fixed with 4% paraformaldehyde at 25°C for 24 h, followed by washing with running water for 0.5 h [20, 21]. The brain was then dehydrated with 75%, 85%, 95%, and anhydrous ethanol for 6 h, 10 h, 4 h, and 2 h, respectively. After permeabilization with xylene I for 20 min and xylene II for 15 min, the brain was embedded in paraffin for 3 h and then cut into 5 *μ*m slices using a microtome (RM2235, Leica Biosystems, Wetzlar, Germany) and roasted at 60°C. After dewaxing with xylene, the slices were stained with hematoxylin and eosin. Random photographs of three fields were acquired using a CX22 light microscope at ×400 magnification (Olympus Corporation, Tokyo, Japan).

### 2.11. Counting of Cryptococcus Neoformans Colonies in the Brain Tissue

The brain tissue was homogenized with normal saline at a ratio of 1 : 1 (g : v). And then, 100 *μ*L homogenate suspension was gradient diluted to concentrations of 1, 0.1, 0.01, and 0.001 g/mL. At each concentration, 10 *μ*L of brain tissue suspension was uniformly applied to yeast extract peptone dextrose medium and cultured in a 30°C incubator for 48 h [22]. Then, the colony count of *cryptococcus neoformans* was performed.

### 2.12. Statistical Analysis

Statistical analysis was conducted by one-way analysis of variance (ANOVA), followed by a Tukey's post hoc test, using SPSS 17.0 (SPSS, Inc., Chicago, IL, USA). Results were presented as the mean ± standard deviation (SD). Statistical differences between two groups were considered significant at *p* < 0.05.

## 3. Results

### 3.1. POS Promotes AMPB Drug Concentration in the Brain Tissue

**Figure 1:**
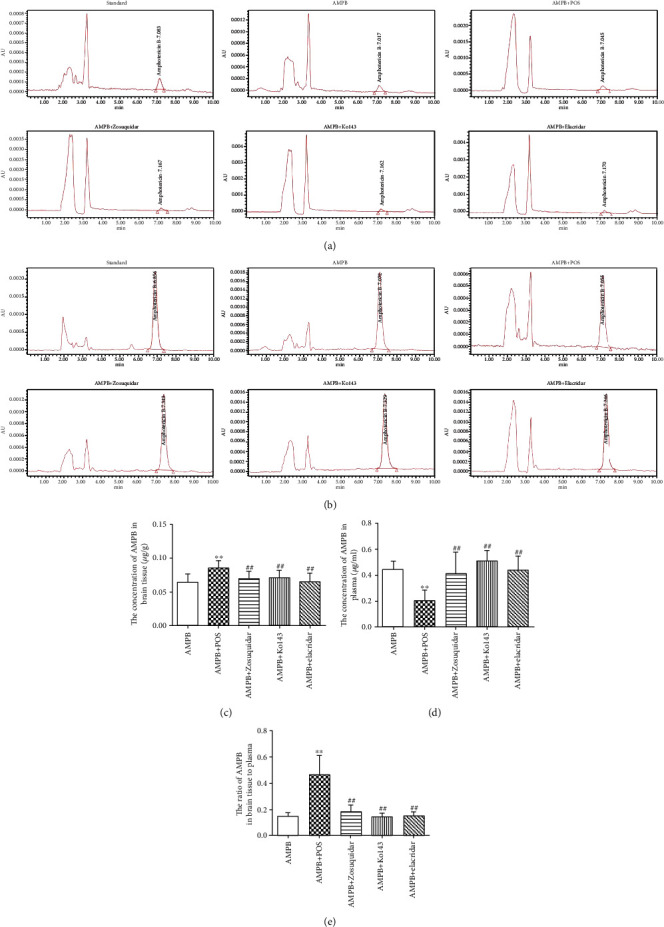
The content of AMPB in the brain tissue and plasma was determined by HPLC. (a) and (b) represent the representative HPLC images of AMPB in the brain tissue and plasma, respectively. (c) and (d) represent the quantitative results of AMPB in the brain tissue and plasma, respectively. (e) represents the quantitative result of the ratio of the brain tissue to plasma AMPB. ^∗∗^*p* < 0.01, compared with the AMPB group; ^##^*p* < 0.01, compared with the AMPB+POS group.

In order to determine whether POS can promote the distribution of AMPB in the brain tissue, the contents of AMPB in the plasma and brain tissue of normal healthy KM mice were detected by HPLC after administering AMPB and AMPB+POS. The results showed that compared with the AMPB group, AMPB+POS can significantly increase the brain tissue concentrations of AMPB (Figures 1(a) and 1(c)*p* < 0.01), while decrease the plasma drug concentration (Figures 1(a) and 1(d)*p* < 0.01), thus increasing the AMPB brain tissue/plasma drug ratio (Figure 1(e)*p* < 0.01), which may partly associate with POS competitive combined with plasma proteins. Further, by adding P-gp inhibitor zosuquidar, BCRP inhibitor Ko143, and a common inhibitor of P-gp and BCRP elacridar, the results showed that compared with the AMPB group, the three inhibitory interventions had no effect on brain tissue concentration, plasma drug concentration, and brain/plasma drug ratio of AMPB, suggesting that the POS promoting brain tissue distribution of AMPB may be independent of BBB-related efflux proteins P-gp and BCRP. At the same time, AMPB may not be a substrate for P-gp and BCRP proteins, which cross the BBB into the brain tissue without the involvement of either.

### 3.2. POS Promotes Plasma Free Drug Concentration of AMPB In Vitro

**Figure 2:**
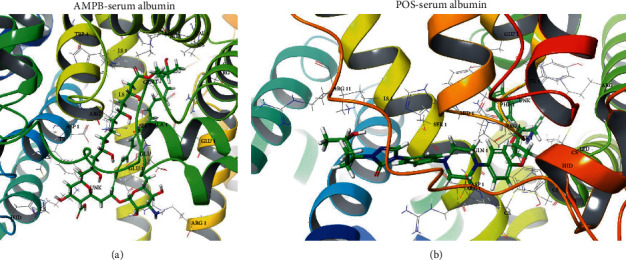
Representative Schrodinger molecular docking diagram of AMPB and POS with serum albumin.

**Figure 3:**
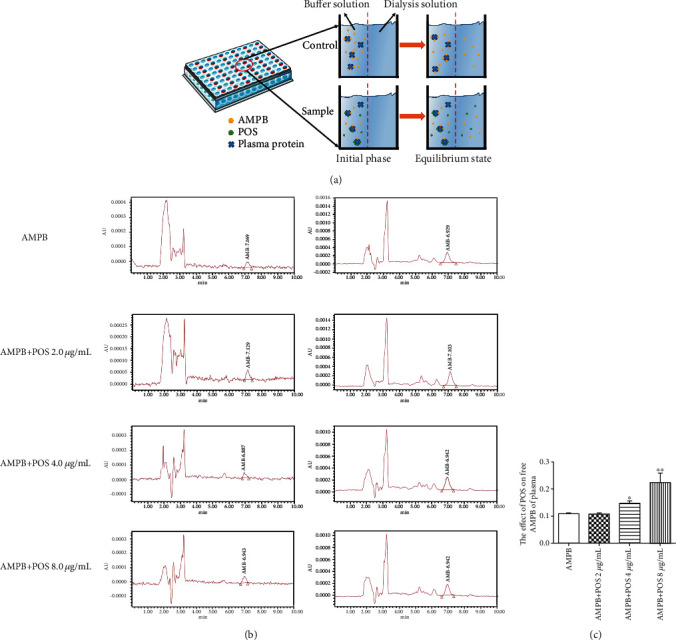
The effect of POS on plasma free AMPB was determined by balanced dialysis. (a) is the principle of balanced dialysis. (b) and (c) represent the representative HPLC images of plasma free AMPB and the quantitative results of plasma free AMPB, respectively. ^∗^*p* < 0.05, compared with the AMPB group; ^∗∗^*p* < 0.01, compared with the AMPB group.

**Figure 4:**
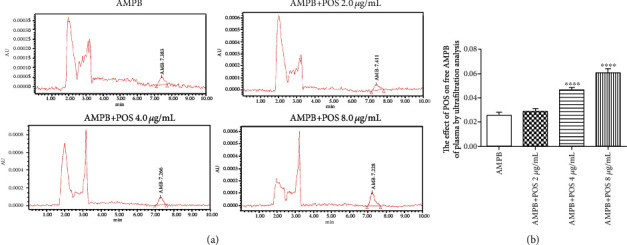
Effect of POS on AMPB free concentration in isolated plasma measured by ultrafiltration analysis. (a) represents the representative HPLC quantitative detection results. (b) represents the quantitative statistical results. *n* = 3, ^∗∗∗∗^*p* < 0.0001, compared with the AMPB group.

To further confirm that POS promoted the distribution of AMPB in the brain tissue possibly due to an increase in its plasma free type drug concentration, we first assessed the binding potential of POS and AMPB to plasma proteins by using Schrodinger's molecular docking. The results in Figure 2 and Table 2 showed that POS and plasma protein had greater binding potential compared with AMPB, with a glide score of 7.718 and AMPB of 6.81, respectively, suggesting that POS and AMPB may competitively bind plasma protein sites and increase the concentration of free AMPB in plasma. Furthermore, we conducted in vitro plasma drug balance dialysis tests by establishing a plasma mixing system containing AMPB or AMPB plus different concentrations of POS (2, 4, and 8 *μ*g/mL). The results showed that 4 (Figures 3(b) and 3(c)*p* < 0.05) and 8 (Figures 3(b) and 3(c)*p* < 0.01) *μ*g/mL POS could prominently elevate the content of AMPB in plasma in vitro, which provided a possibility for improving the distribution of AMPB in the brain tissue. Similarly, the ultrafiltration analysis in Figure 4 further demonstrated that 4 and 8 *μ*g/mL (*p* < 0.0001) POS could potentially increase the plasma concentration of free AMPB. The above results suggest that the mechanism by which POS promotes plasma free AMPB concentration may be related to its competitive binding to plasma protein sites.

### 3.3. POS Has no Effect on AMPB Uptake by BCECs In Vitro

**Figure 5:**
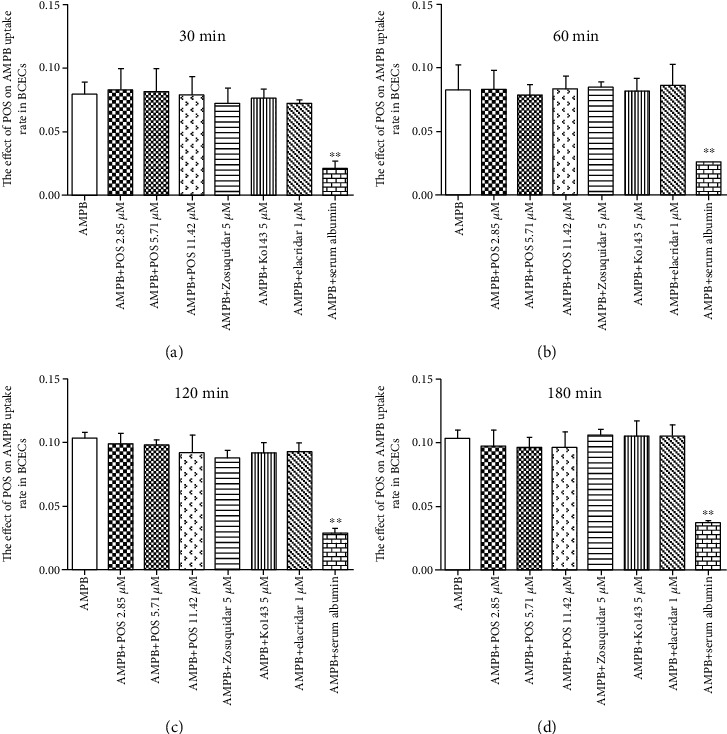
Effects of POS on AMPB uptake by BCECs at 30, 60, 120, and 180 min. ^∗∗^*p* < 0.01, compared with the AMPB group.

We further demonstrated that the distribution of POS promoting AMPB in the brain tissue may be independent of the BBB-related efflux proteins P-gp and BCRP, while increasing the plasma free drug concentration of AMPB through in vitro uptake of AMPB by mice BCECs. The results in Figure 5 showed that compared with the AMPB group, POS (2.85, 5.71 and 11.42 *μ*M) in different concentrations had no significant effect on AMPB uptake by BCECs with the extension of culture time. Meanwhile, compared with the AMPB group, the P-gp inhibitor zosuquidar, the BCRP inhibitor Ko143, and the joint inhibitor elacridar of P-gp and BCRP had no significant effect on the uptake of AMPB by BCECs in the three groups. Nevertheless, compared with the AMPB group, serum albumin intervention strikingly inhibited the uptake of AMPB by BCECs at 30 (*p* < 0.01), 60 (*p* < 0.01), 120 (*p* < 0.01), and 180 (*p* < 0.01) min. Representative HPLC images are shown in Figure S1. The above results confirmed again that POS promoted the distribution of AMPB in brain tissues by increasing the plasma free drug concentration of AMPB by competitively binding to the plasma protein site, rather than by inhibiting the function of the BBB efflux proteins P-gp and BCRP.

### 3.4. POS Enhances the Efficacy of AMPB in Mice with Cryptococcal Meningitis

**Figure 6:**
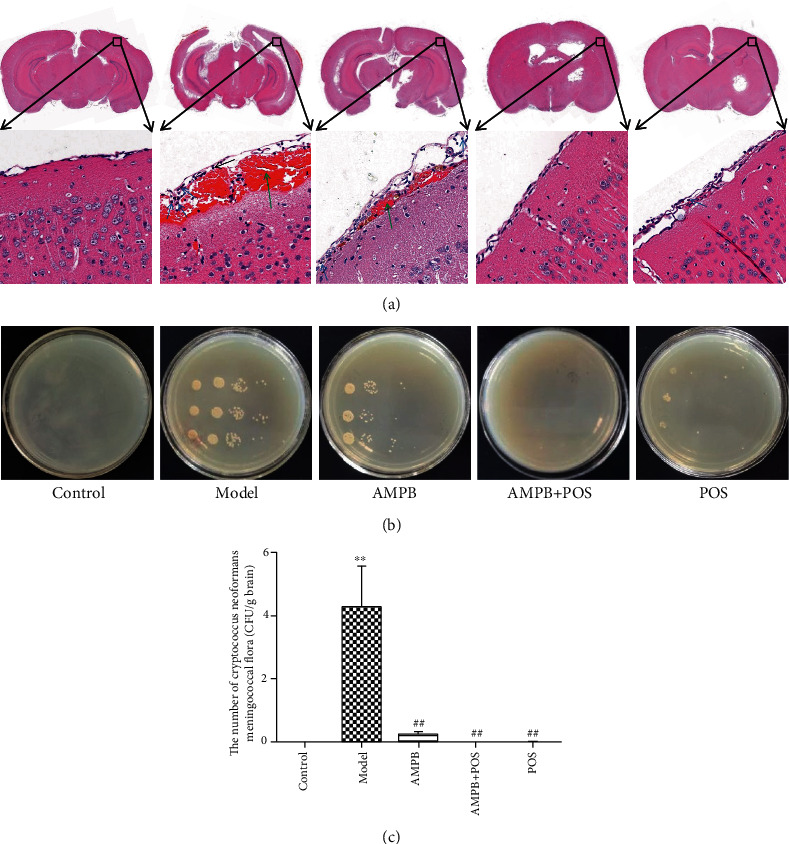
The effect of POS on AMPB in the treatment of cryptococcal meningitis in mice. (a) represents that AMPB combined with POS significantly improved cerebral cortex pathological injury in mice with cryptococcal meningitis. Blue arrow: neutrophils; black arrow: lymphocytes; green arrow: bleeding. (b) indicates that AMPB combined with POS markedly restricts the proliferation of *cryptococcus neoformans* in meningitis mice. (c) represents the statistical result of the number of cryptococcus neoformans in the mouse brain. ^∗∗^*p* < 0.01, compared with the control group; ^##^*p* < 0.01, compared with the model group.

The results of H&E staining in Figure 6(a) showed that, compared with the control group, there were more inflammatory cell infiltrations in the cortical neurons of the model group, evidenced by increased neutrophils and lymphocytes. Meanwhile, severe bleeding occurred in the model group. However, AMPB, AMPB+POS, and POS treatment could improve the above pathological injuries, among which the AMPB+POS group had the best effect.

Similarly, colony culture results of *cryptococcus neoformans* showed a noteworthy increase in the number of *cryptococcus neoformans* in the model group compared with the control group (Figure 6(b) and 6(c)*p* < 0.01). While compared with the model group, the AMPB group (*p* < 0.01), the AMPB+POS group (*p* < 0.01), and the POS group (*p* < 0.01) could evidently inhibit the number of *cryptococcus neoformans*, among which the AMPB+POS group had the best inhibitory effect. Compared with the AMPB group, the AMPB+POS group showed a statistically significant difference in the amount of inhibition of *cryptococcus neoformans* (*p* < 0.01). The above results of H&E pathological staining of the mouse brain tissue and colony culture of *cryptococcus neoformans* proved that POS could enhance the efficacy of AMPB in the treatment of CNS.

## 4. Discussion

Previous studies have shown that AMPB and POS alone, or in combination with other antifungal agents, are effective in the treatment of cryptococcal meningitis [23–25]. However, the BBB has an efflux effect on both, so the reduced distributed concentration in the brain makes the clinical treatment of cryptococcal meningitis less effective. Although there is an optimized delivery system designed to enhance AMPB's ability to cross the BBB, its potential blood and multitissue toxicity has not been systematically evaluated [26]. On the other hand, it has not been reported whether POS can promote the distribution of AMPB in the brain tissue. Our in vivo studies have shown that the combination of AMPB and POS can reduce the level of total AMPB in plasma, possibly due to increased distribution in the brain tissue. Similarly, HPLC results showed that POS significantly increased the concentration of AMPB in the brain tissue. For drugs with higher plasma protein binding rates, such as AMPB and POS, which have plasma protein binding rates of 90% and 98%, respectively, small changes in plasma protein binding rates can cause significant changes in plasma free drug concentrations [27]. In this study, Schrodinger's molecular docking results suggested that POS promotion of the AMPB brain tissue distribution may be partly related to their competition for plasma protein binding sites. We further confirmed POS's ability to compete with AMPB to bind plasma proteins through in vitro plasma balance dialysis and ultrafiltration analysis, thereby increasing the plasma free drug concentration of AMPB, which provided the potential for more AMPB to enter the brain tissue. In general, our animal studies and plasma balance dialysis results suggest that POS can increase the plasma free drug concentration and brain tissue distribution of AMPB.

Evidence suggests that P-gp in the BBB prevents AMPB from entering the brain tissue; however, whether AMPB is a substrate of P-gp is still controversial [12, 13]. In animal experiments, by adding P-gp inhibitor zosuquidar, BCRP inhibitor Ko143, and a common inhibitor of P-gp and BCRP elacridar, the results showed that all three inhibitors had no obvious effect on the concentration of AMPB in the brain tissue of mice, suggesting that the mechanism of the distribution of AMPB in the brain tissue may be independent of the expression of P-gp and BCRP proteins. Further, in vitro BCEC intake experimental results confirmed that POS promoted the brain tissue distribution of AMPB, partly through a competitive combination of plasma protein and increasing the content of plasma free AMPB, rather than by inhibiting BBB P-gp and BCRP proteins, which were consistent with previous conclusions of AMPB in vitro. Finally, we demonstrated in a mouse model of CNM that POS notably enhanced the efficacy of AMPB in the treatment of meningitis, as shown by the regular array of cortical neurons, suppressed inflammatory damage as well the number of *Cryptococcus neoformans*.

Inevitably, there are also obvious shortcomings in our experiment. First, we lack direct evidence that AMPB is not a substrate for P-gp and BCRP proteins. Overexpressed, knocked out or silenced P-gp and BCRP genes may be the most powerful tool to investigate the interaction between AMPB with P-gp and BCRP [28]. At the same time, PET or brain-targeted mass spectrometry imaging technology is also the most intuitive choice for the interpretation of the drug brain tissue distribution and drug-target action mode [29]. Secondly, we lack effective models to simulate the complex structure and function of the BBB in the physiological environment. Next, we plan to construct a three-dimensional BBB model using in vitro microminiaturized chips and integrate microfluidic and mass spectrometry imaging techniques to uncover whether AMPB's crossing the BBB is related to P-gp and BCRP proteins [30]. Finally, we strive to elucidate the underlying mechanism by which POS promotes the distribution of AMPB in brain tissue.

## 5. Conclusions

In summary, we demonstrate for the first time that POS can promote the distribution of AMPB in the brain tissue through in vitro and in vivo experiments and thus has a stronger therapeutic effect on cryptococcal meningitis. The underlying mechanism may be related to POS's competitive binding to plasma proteins rather than the inhibition of BBB-related drug efflux proteins, P-gp and BCRP. However, the clinical efficacy of POS in promoting the AMPB brain tissue distribution in the treatment of cryptococcal meningitis needs to be further probed.

## Figures and Tables

**Table 1:** The group information of POS on AMPB free concentration in *in vitro* plasma by ultrafiltration analysis.

Groups	Base solution	AMPB(*μ*g/mL)	POS(*μ*g/mL)
1	Plasma	2.0	0.0
2	Plasma	2.0	2.0
3	Plasma	2.0	4.0
4	Plasma	2.0	8.0

**Table 2:** Lowest binding energy for the ligand–protein interactions detected by Glide molecular docking.

Protein	PDB ID	Compounds	PubChem CID	Glide score	Glide H-bond	Glide evdw
Serum albumin	1N5U	AMPB	5280965	-6.81	-0.441	-56.377
		POS	468595	-7.718	-0.185	-60.975

## Data Availability

The raw data supporting the conclusions of this article will be made available by the corresponding author, without undue reservation, to any qualified researcher.

## Supplementary Materials

Supplementary Materials Supplemental Figure 1: representative HPLC profiles of POS on AMPB uptake by BCECs at 30 (a), 60 (b), 120 (c), and 180 (d) min.
