# Supplementary material for: A Novel Cryptococcal Meningitis Therapy: The Combination of Amphotericin B and Posaconazole Promotes the Distribution of Amphotericin B in the Brain Tissue
Source: Biomed Res Int. 2020 Nov 29;2020:8878158. doi: 10.1155/2020/8878158 (PMC7719495; doi:10.1155/2020/8878158)
Supplement: Supplementary Materials — Supplemental Figure 1: representative HPLC profiles of POS on AMPB uptake by BCECs at 30 (a), 60 (b), 120 (c), and 180 (d) min. [file 8878158.f1.docx]

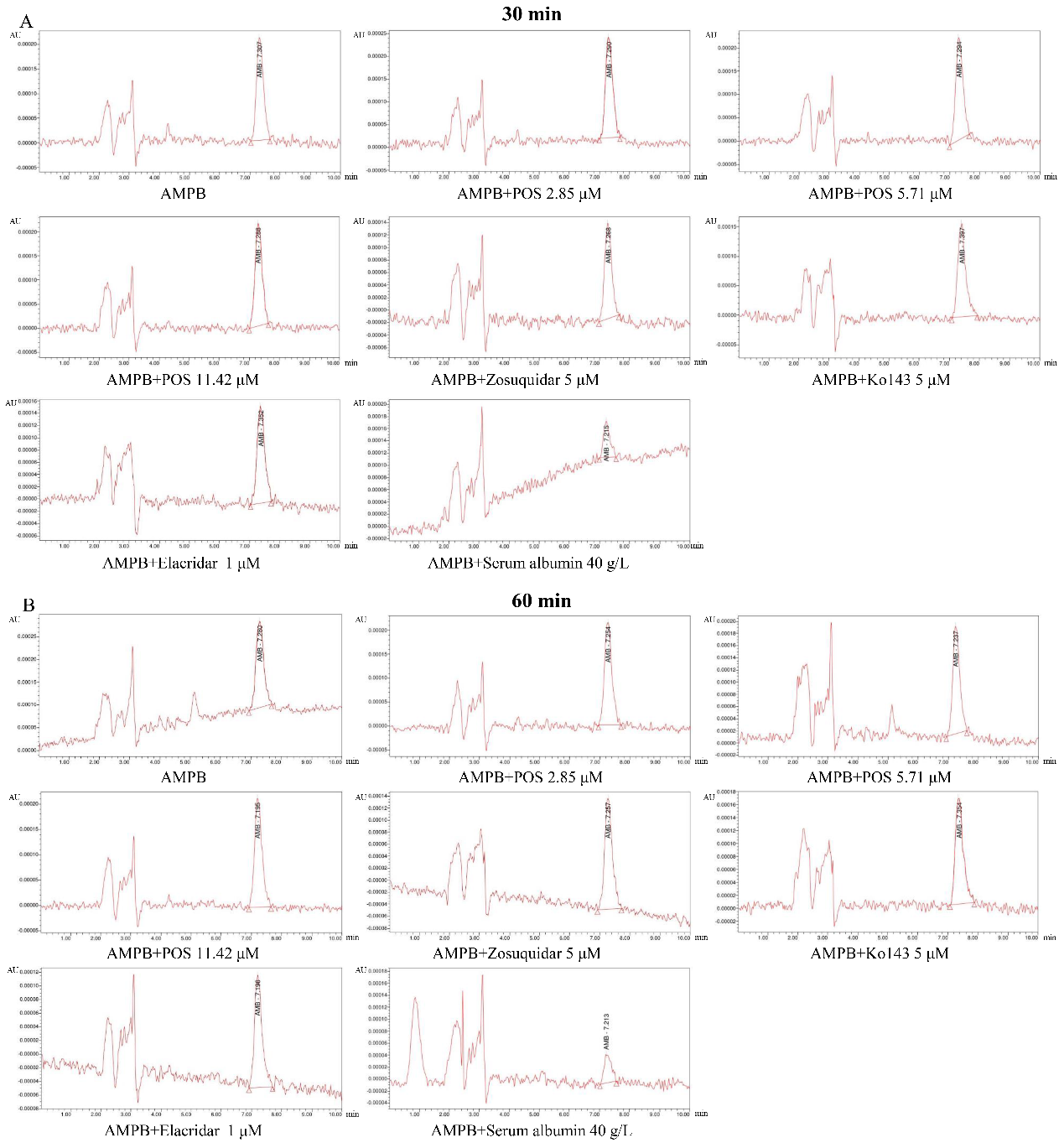


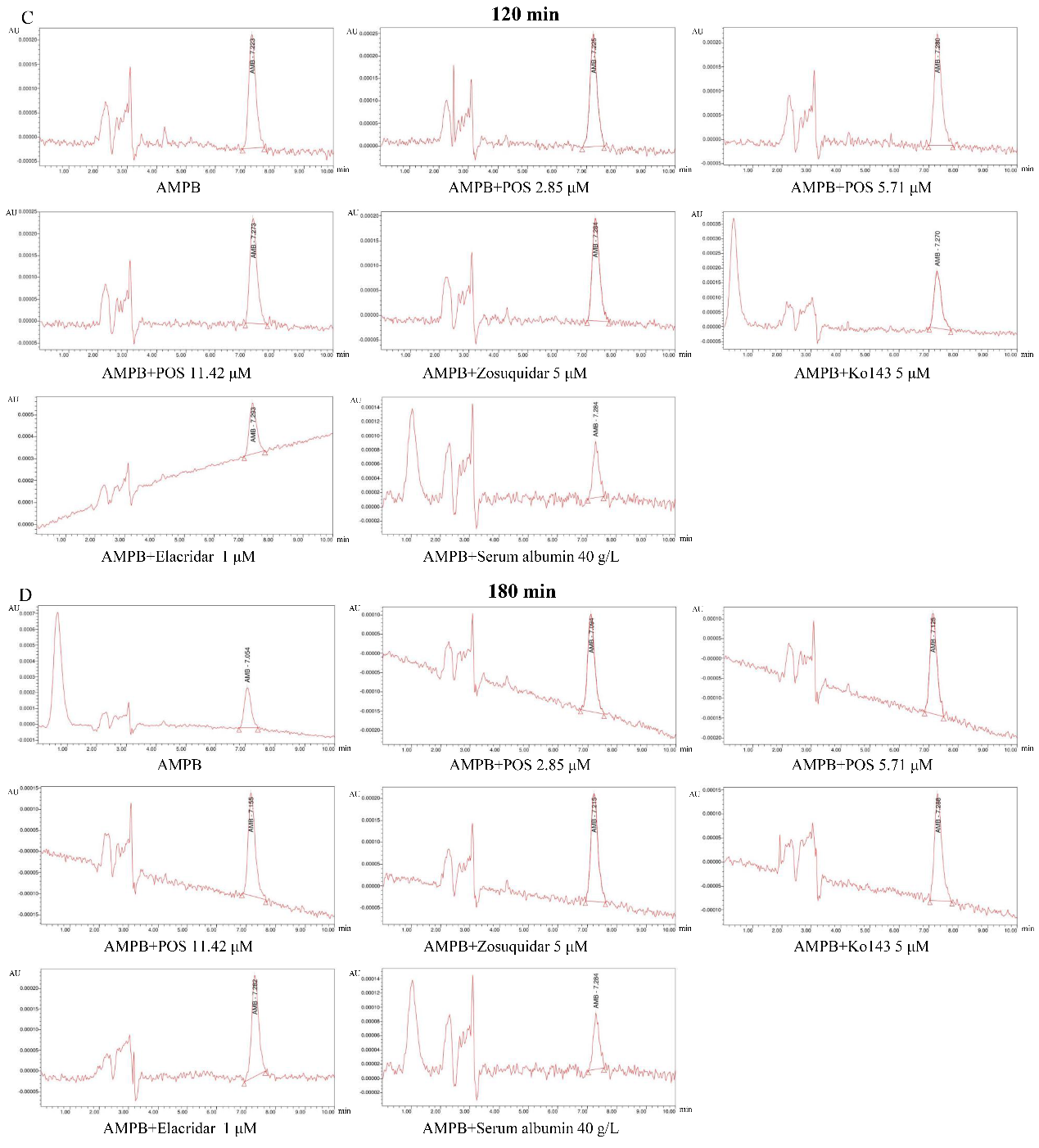
 Supplemental F_IGURE_ 1 Representative HPLC profiles of POS on AMPB uptake by BCECs at 30 (A), 60 (B), 120 (C) and 180 (D) min.
